# Hepaticojejunostomy Insufficiency-Associated Arterial Hemorrhage in Patients After Pancreatic Surgery

**DOI:** 10.3390/jcm15082900

**Published:** 2026-04-10

**Authors:** Torsten Herzog, Marcus-Thomas Skrobisch, Ahmed Abdelsamad, Waldemar Uhl, Orlin Belyaev, Ilgar Aghalarov, Jennifer Herzog-Niescery

**Affiliations:** 1 Department of General and Visceral Surgery, Katholisches Klinikum Bochum, St. Josef Hospital, Ruhr University Bochum, Gudrunstraße 56, 44791 Bochum, Germany; torsten.herzog@knappschaft-kliniken.de (T.H.); m.skrobisch@web.de (M.-T.S.); w.uhl@klinikum-bochum.de (W.U.); o.belyaev@klinikum-bochum.de (O.B.); i.aghalarov@klinikum-bochum.de (I.A.); 2 Department of General and Visceral Surgery, Knappschaft-Kliniken Vest GmbH, Dorstener Str. 151, 45657 Recklinghausen, Germany; ahmed.abdelsamad@knappschaft-kliniken.de; 3 Department of Anesthesiology, Katholisches Klinikum Bochum, St. Josef Hospital, Ruhr University Bochum, Gudrunstraße 56, 44791 Bochum, Germany

**Keywords:** postpancreatectomy hemorrhage, hepaticojejunostomy insufficiency, arterial erosion, biliary leakage, interventional angiography

## Abstract

**Background**: Postoperative hemorrhage is a severe complication after pancreatic surgery. While bleeding related to pancreatic fistula is well characterized, hemorrhage secondary to biliary leakage remains poorly understood. This study investigates the incidence, associated factors, clinical course, and outcomes of hepaticojejunostomy insufficiency-associated arterial hemorrhage (HIAA). **Methods**: This retrospective single-center study included 1413 patients who underwent pancreatic surgery with hepaticojejunostomy between 2004 and 2014. Demographics, underlying disease, surgical procedures, postoperative complications, management strategies, and outcomes were analyzed. **Results**: HIAA occurred in 13 patients (0.9%), accounting for one third of all erosion-related hemorrhages. The median onset was 16 days postoperatively, and 77% were preceded by sentinel bleeding. Completion pancreatectomy and sepsis were significantly associated with HIAA. The right hepatic artery was the most frequent bleeding source. Primary interventional angiography achieved hemostasis in 62.5% of patients, while 61.5% required surgical revision. Thirty- and ninety-day mortality rates were 15.4% and 30.8%, respectively, compared with 2.1% and 3.7% in the overall cohort. **Conclusions**: HIAA is a rare but highly lethal complication after pancreatic surgery. It represents a distinct clinical entity characterized by delayed onset, frequent sentinel bleeding, an association with sepsis and completion pancreatectomy, and markedly increased mortality. Early recognition, prompt imaging, and an interventional-first strategy are essential to improve outcomes.

## 1. Introduction

Pancreatic diseases such as chronic pancreatitis and pancreatic ductal adenocarcinoma continue to be associated with a poor prognosis, with surgical resection representing the only potentially curative treatment option for most patients. Despite advances in surgical technique and perioperative management, pancreatic surgery remains burdened by substantial morbidity and mortality. This is largely attributable to the complex anatomy of the pancreas, its close proximity to major arterial and venous structures, the technically demanding reconstruction, and the corrosive effects of pancreatic and biliary secretions on surrounding tissues [1,2].

Postoperative fistula formation and hemorrhagic complications are among the most severe and potentially life-threatening adverse events following pancreatic surgery [3]. Postoperative pancreatic fistula, as defined by the International Study Group of Pancreatic Surgery and updated in 2016, occurs in approximately 10–20% of patients after pancreatoduodenectomy and is a well-established risk factor for secondary arterial erosion and delayed hemorrhage [3,4,5]. In contrast, postoperative biliary fistula is reported in fewer than 10% of cases in high-volume centers and is often considered clinically less relevant when occurring in isolation [6]. Nevertheless, both pancreatic and biliary leaks may trigger local inflammation, sepsis, and progressive tissue damage, ultimately leading to organ failure and death [1,2,3,4,5,6].

Post-pancreatectomy hemorrhage remains a major contributor to postoperative morbidity and mortality, with reported incidences ranging from 3% to 10% depending on the surgical procedure and definition [3]. According to the International Study Group of Pancreatic Surgery, hemorrhage is classified as early or delayed, with delayed post-pancreatectomy hemorrhage (DPH) typically resulting from local inflammatory processes, intra-abdominal abscess formation, or anastomotic leakage [3]. DPH often manifests as a so-called “sentinel bleed,” and timely diagnosis and intervention are crucial, as mortality rates of severe cases may reach up to 50% [7,8].

While the association between postoperative pancreatic fistula and DPH is well documented, the role of biliary leakage in the development of postoperative hemorrhage remains poorly defined. Several studies have identified postoperative pancreatic fistula as an independent risk factor for arterial erosion, most commonly involving the gastroduodenal or hepatic arteries [7,8,9,10]. Bile, however, exhibits detergent and proteolytic properties that may similarly impair vascular integrity and promote tissue necrosis. Despite this theoretical risk, hemorrhagic complications specifically attributable to hepaticojejunostomy (HJ) insufficiency have received limited attention. Most available studies have focused on pancreatic fistula-associated bleeding or have analyzed biliary leaks only in the context of combined anastomotic failure, precluding an assessment of their independent impact [8,9,10,11].

To date, no comprehensive analysis has specifically addressed DPH secondary to isolated biliary fistula following pancreatic surgery. This knowledge gap is clinically relevant, as such events may be underestimated or misclassified in the postoperative course. Improved understanding of associated factors, clinical presentation, and outcomes may facilitate earlier recognition and more effective management of this rare but potentially fatal complication.

The present study therefore aimed to investigate the incidence of delayed postoperative hemorrhage associated with postoperative biliary fistula after pancreatic surgery in a high-volume tertiary referral center. In addition, potential associated factors, perioperative management strategies, and patient outcomes were systematically analyzed to better characterize this complication and identify opportunities for prevention and early intervention.

## 2. Materials and Methods

This retrospective single-center investigation was conducted at a German university hospital. The study was approved by the institutional review board of the Ruhr-University Bochum (No. 16-5591) and performed in accordance with the Declaration of Helsinki as revised in 2013. All patients provided written informed consent for surgery and data collection.

### 2.1. Patient Cohort

The cohort of this study included all consecutive patients who underwent pancreatic surgery with primary HJ at the Department of General and Visceral Surgery, St. Josef Hospital Bochum, between 1 January 2004, and 31 December 2014, regardless of the underlying disease. Patients who developed postoperative HJ insufficiency complicated by erosion-related hemorrhage were identified and analyzed. In addition to the overall cohort analysis, a predefined subgroup analysis was performed among patients with HJ insufficiency, comparing those who developed arterial hemorrhage to those without hemorrhage, in order to identify factors associated with progression to HIAA.

### 2.2. Data Collection

Clinical data were retrieved from electronic medical records (ORBIS, Agfa HealthCare) and archived paper charts. Data were pseudonymized and entered into a dedicated database for analysis. Extracted variables included demographic data, preoperative clinical and laboratory findings, imaging results, intraoperative parameters, microbiological and histopathological findings, as well as postoperative complications and management strategies.

### 2.3. Surgical Procedures and Perioperative Management

All pancreatic resections requiring biliary reconstruction were included. Surgical procedures comprised total pancreatectomy, classic pancreatoduodenectomy (Kausch–Whipple), pylorus-preserving pancreatoduodenectomy, non-resective biliary bypass procedures with or without gastrojejunostomy (double bypass), completion pancreatectomy, and revisional procedures including redo-pancreaticojejunostomy or redo-HJ. Hepaticojejunostomy was performed in a standardized fashion as an end-to-side, single-layer anastomosis using interrupted 5-0 polydioxanone sutures. In patients with small-caliber (<5 mm) or fragile bile ducts, external biliary drainage with a T-tube was applied at the surgeon’s discretion.

### 2.4. Definitions of Fistula, Hemorrhage, and Complications

Postoperative biliary fistula was defined according to the International Study Group of Liver Surgery as bilious drainage fluid or intra-abdominal collections with a bilirubin concentration at least three times higher than the corresponding serum value, occurring on or after postoperative day three [9].

Postoperative pancreatic fistula was defined according to the International Study Group of Pancreatic Surgery criteria as drainage fluid with an amylase concentration exceeding three times the upper normal serum value and graded as A–C based on clinical impact.

DPH was defined and classified according to the International Study Group of Pancreatic Surgery criteria based on timing, location, severity, and clinical impact. Clinically relevant hemorrhage included grade B events (sentinel bleeding) and grade C events (life-threatening hemorrhage) [10]. Sentinel bleeding was defined as minor intra- or extraluminal bleeding occurring more than 24 h after surgery without hemodynamic compromise but preceding severe hemorrhage [11].

Other postoperative complications, including sepsis, delayed gastric emptying, intra-abdominal abscess, wound infection, lymphatic fistula, cholangitis, and burst abdomen, were recorded and defined according to established international criteria.

### 2.5. Diagnosis and Management of Hemorrhage

In hemodynamically stable patients, contrast-enhanced CT angiography followed by selective interventional angiography was performed to localize the bleeding source. Interventional radiological treatment included coil embolization or placement of covered stents. Hemodynamically unstable patients or those with failed interventional therapy underwent immediate surgical revision.

### 2.6. Outcome Measures

The primary outcome was the occurrence of DPH associated with biliary fistula. Secondary outcomes included timing of sentinel bleeding, hemoglobin nadir, transfusion requirements, length of hospital stay, postoperative morbidity, and 30-day and 90-day mortality.

### 2.7. Statistical Analysis

Statistical analysis was performed using SPSS software (version 22.0, IBM Corp., Ehningen, Germany). Categorical variables were summarized as absolute and relative frequencies. Continuous variables were expressed as mean ± standard deviation or median with range, as appropriate. Normal distribution was assessed using histograms, Kolmogorov–Smirnov, and Shapiro–Wilk tests. Comparisons between groups were performed using the unpaired *t* test or Mann–Whitney U test for continuous variables and the chi-square test or Fisher’s exact test for categorical variables. A two-sided *p*-value < 0.05 was considered statistically significant.

## 3. Results

**Figure 1:**
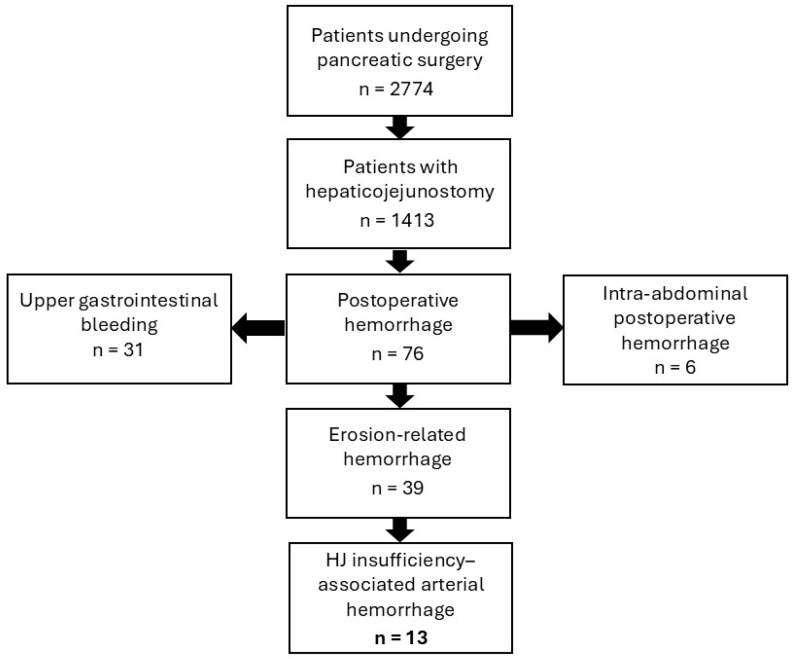
Flow chart illustrating patient selection and identification of hepaticojejunostomy insufficiency-associated arterial hemorrhage following pancreatic surgery. The incidence was 0.9% in this cohort. HJ = hepaticojejunostomy.

Between January 2004 and December 2014, 2774 patients underwent pancreatic surgery at our institution. A hepaticojejunostomy (HJ) was performed in 1413 patients (50.9%), constituting the study cohort. Postoperative hemorrhage occurred in 76 patients (5.4%), of which 39 cases were erosion-related. Thirteen hemorrhages were attributed to HJ insufficiency, resulting in an overall incidence of hepaticojejunostomy insufficiency-associated arterial hemorrhage (HIAA) of 0.9% (13/1413) (Figure 1).

### 3.1. Patient Characteristics

The mean age of patients with HJ was 62.3 ± 12.8 years (median 64, range 6–91), and 57.5% were male. Patients in the HIAA group were slightly younger, with a mean age of 57.0 ± 18.5 years (median 58, range 6–78), and 69.2% were male. Neither age nor sex differed significantly between groups (*p* = 0.332 and *p* = 0.391, respectively).

In patients without HIAA, surgery was performed for malignant disease in 64.9% and for benign conditions in 35.1%. Pancreatic ductal adenocarcinoma was the most frequent indication, followed by cholangiocarcinoma. Chronic pancreatitis was the most common benign indication for surgery (Table 1). Among patients with HIAA, benign disease was more frequent than malignant disease, with chronic pancreatitis representing the most common indication.

The surgical procedures are also summarized in Table 1. Among patients who developed HIAA, pylorus-preserving pancreatoduodenectomy was the most frequent index procedure (38.5%). Completion pancreatectomy was significantly associated with the occurrence of HIAA, with 30.8% of patients undergoing this procedure developing hemorrhage related to HJ insufficiency (*p* < 0.001).

### 3.2. Postoperative Morbidity

Postoperative morbidity occurred in 40.2% of all patients (568/1413). The most frequent minor complications were delayed gastric emptying (11.8%; 167/1413), wound infection (6.3%; 89/1413), lymphatic fistula (4.6%; 65/1413), and intra-abdominal abscess (2.7%; 38/1413). Differences between patients with and without HIAA were not statistically significant (all *p* > 0.256). Major complications are listed in Table 2.

Erosion hemorrhage was significantly associated with HJ insufficiency, PJ insufficiency, cholangitis, and sepsis (all *p* < 0.001; Table 2).

### 3.3. HIAA: Characteristics, Management, and Outcome

**Figure 2:**
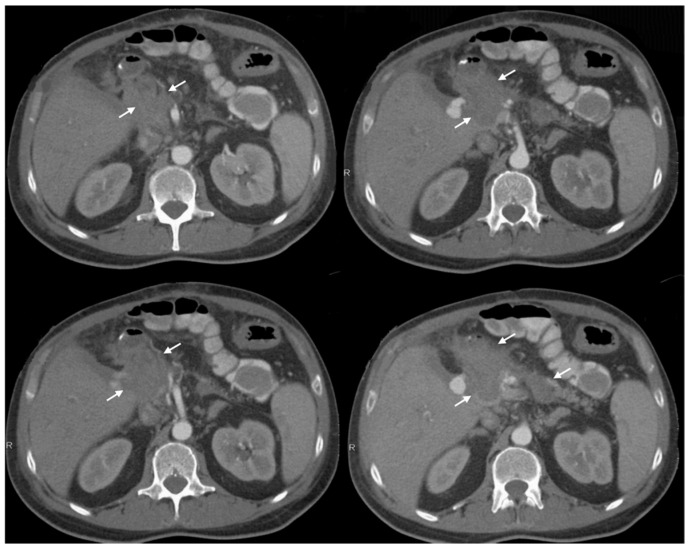
CT Angiography of Right Hepatic Artery Pseudoaneurysm in HIAA. Contrast-enhanced CT imaging of a patient with hepaticojejunostomy insufficiency-associated arterial hemorrhage demonstrating a large hematoma (white arrows) secondary to pseudoaneurysmal bleeding of the right hepatic artery. Due to severe stenosis at the origin of the celiac trunk, endovascular intervention was not feasible and immediate surgical management was required.

HIAA occurred at a mean of 19.9 ± 12.5 days postoperatively (median 16 days, range 5–53). The right hepatic artery was the most frequent source of bleeding (61.5%; n = 8/13), followed by the left hepatic artery (15.4%; n = 2/13) (Figure 2). Sentinel bleeding preceded definitive hemorrhage in 76.9% (n = 10/13) of patients.

Based on International Study Group of Pancreatic Surgery criteria, no grade A hemorrhage (clinically irrelevant bleeding) occurred. Grade B hemorrhage, necessitating therapeutic intervention, was observed in 38.5% (n = 5/13) of patients, while 61.5% (n = 8/13) experienced grade C hemorrhage, defined by severe bleeding with hemodynamic compromise, organ failure, or fatal outcome. The mean hemoglobin nadir was 7.5 ± 0.7 g/dL. Deceased patients required significantly more red blood cell transfusions than survivors (23.3 ± 10.8 vs. 12.1 ± 5.2 units, *p* = 0.026). Patients with grade C hemorrhage required significantly more transfusions than those with grade B hemorrhage (*p* = 0.038).

**Figure 3:**
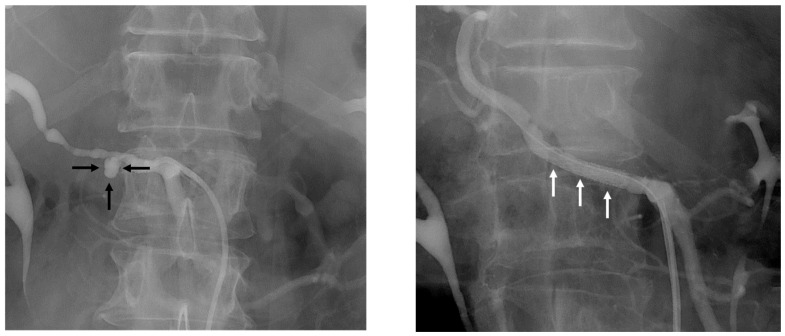
Endovascular management of right hepatic artery pseudoaneurysm. Angiographic visualization of a right hepatic artery pseudoaneurysm (black arrows) before (**left**) and after covered stent (white arrows) implantation (**right**).

Primary interventional angiography was performed in 61.5% (n = 8/13) of patients, resulting in successful hemostasis in 62.5% (n = 5/8), achieved by covered stent placement or coil embolization (Figure 3).

Surgical revision was ultimately required in 61.5% (n = 8/13) of patients, including those without primary interventional management, and those with failed angiographic hemostasis. Among patients undergoing relaparotomy, T-tube drainage was performed in 87.5% (n = 7/8). Hemostasis was achieved surgically in 50% (n = 4/8) of cases, and patients treated with T-tube placement could be discharged without further complications in most cases.

Mortality differed according to treatment modality, with lower mortality observed after successful primary interventional management compared with patients requiring surgical revision or combined approaches. Thirty-day mortality in the overall cohort was 2.1% (n = 29/1413), and 90-day mortality was 3.7% (n = 92/1413). In contrast, patients with HIAA exhibited a markedly increased 30-day mortality of 15.4% (n = 2/13) and a 90-day mortality of 30.8% (n = 4/13; *p* < 0.02 and *p* = 0.010, respectively). In the overall cohort, deaths were most commonly attributable to multiorgan failure (2.3%; n = 32/1413), followed by cardiovascular failure (0.5%; n = 7/1413), respiratory failure (0.5%; n = 7/1413), acute liver failure (0.3%; n = 4/1413), and hepatorenal syndrome (0.2%; n = 3/1413); isolated cases were due to tumor cachexia or intracerebral hemorrhage. In the HIAA group, deaths were directly related to hemorrhagic shock or consecutive multiorgan failure.

### 3.4. Factors Associated with HIAA in Patients with HJ Insufficiency

To further explore factors associated with progression to arterial hemorrhage, a subgroup analysis was performed comparing patients with HIAA (n = 13) to those with isolated hepaticojejunostomy insufficiency without hemorrhage (n = 64). No significant differences were observed between patients with and without HIAA in terms of age (*p* = 0.226), sex (*p* = 1.000), or underlying diagnosis (*p* = 0.166). In contrast, completion pancreatectomy was significantly associated with the occurrence of HIAA (*p* < 0.001), and sepsis occurred more frequently in patients with HIAA than in those with isolated HJ insufficiency (*p* = 0.010).

Overall mortality among patients with HJ insufficiency was 18.2% (14/77). Mortality was higher in patients with HIAA compared with those without hemorrhage (30.8% [4/13] vs. 15.6% [10/64]); however, this difference did not reach statistical significance (*p* = 0.239).

## 4. Discussion

Postoperative hemorrhage remains one of the most devastating complications following pancreatic surgery. While DPH is well characterized in the context of pancreatic fistula, bleeding secondary to biliary leakage has received far less attention and is usually reported only as part of heterogeneous bleeding cohorts [12,13]. In this study, HIAA occurred in fewer than 1% of patients undergoing pancreatic surgery with HJ, yet accounted for one third of all erosion-related arterial hemorrhages. Importantly, progression from isolated biliary leakage to arterial hemorrhage was associated with a substantial increase in mortality. These findings suggest that HIAA may represent a clinically distinct and highly severe subtype of DPH rather than merely an extension of biliary complications.

Bile acids are amphipathic molecules with pronounced detergent properties that disrupt cellular membranes and promote tissue necrosis, while fibrinolytic proteins present in human bile accelerate clot dissolution and impair local hemostasis [14,15]. In the setting of persistent biliary leakage and superimposed infection, these mechanisms may progressively weaken the arterial wall, ultimately leading to pseudoaneurysm formation and rupture. Comparable processes have been described for pancreatic fistula-associated hemorrhage, suggesting overlapping but pathophysiologically distinct pathways depending on the type of anastomotic failure [16].

Completion pancreatectomy showed the strongest association with HIAA in our cohort, both in the overall analysis and among patients with HJ insufficiency. This observation should be interpreted with caution given the limited number of events but may reflect the complex surgical and inflammatory conditions in which completion pancreatectomy is typically performed. These conditions may predispose to biliary anastomotic breakdown and simultaneously facilitate septic arterial erosion in adjacent vascular structures [16]. In contrast, demographic variables and underlying pathology were not associated with HIAA, underscoring that local and procedural factors outweigh patient-related characteristics. The significantly higher incidence of sepsis among patients who developed hemorrhage further supports the hypothesis that infection may play an important role in the progression from biliary leakage to arterial erosion.

Clinically, HIAA predominantly presented as delayed hemorrhage, typically occurring approximately three weeks after surgery. In more than three quarters of patients, bleeding was heralded by sentinel hemorrhage. This finding is of particular relevance, as sentinel bleeding is a well-established warning sign of impending arterial rupture and should prompt immediate diagnostic escalation rather than conservative observation [17,18,19]. In patients with known HJ insufficiency, sentinel bleeding should therefore be interpreted as a vascular emergency.

The right hepatic artery was the most common bleeding source, reflecting its close anatomical relationship to the HJ. Accordingly, contrast-enhanced computed tomography with an angiographic phase represents the diagnostic modality of choice, allowing precise localization of the bleeding site and enabling rapid therapeutic decision-making [20,21].

**Figure 4:**
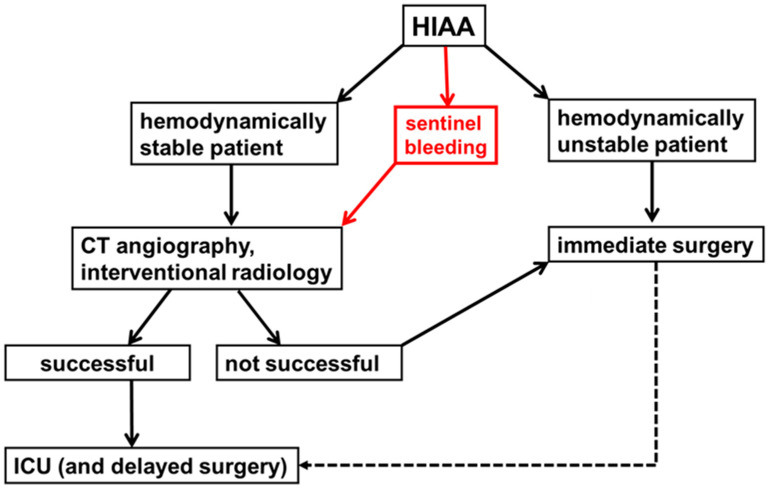
Proposed management algorithm for hepaticojejunostomy insufficiency-associated arterial hemorrhage (HIAA). Management is guided primarily by hemodynamic stability and the need for control of the underlying septic focus. In hemodynamically stable patients, contrast-enhanced imaging followed by primary endovascular intervention is recommended. In unstable patients, immediate surgical exploration with hemostasis and revision of the hepaticojejunostomy, including T-tube drainage, is required. Even after successful endovascular treatment, additional surgical revision may be necessary to achieve definitive source control.

Management of HIAA remains challenging and frequently necessitates a multimodal approach based on hemodynamic status and control of the underlying septic focus. In patients with HJ insufficiency without active bleeding, conservative management is feasible if adequate drainage is ensured. However, once arterial hemorrhage occurs, rapid bleeding control becomes the primary priority. Then, hemodynamic stability is the key determinant of the initial strategy. In stable patients, contrast-enhanced imaging followed by endovascular intervention should be considered first-line therapy. In our cohort, primary interventional angiography achieved successful hemostasis in nearly two thirds of patients, using either coil embolization or covered stent implantation. These findings are consistent with growing evidence that endovascular therapy represents the preferred first-line treatment for DPH, offering rapid bleeding control while avoiding re-laparotomy in critically ill patients [16,22]. In unstable patients, immediate surgical exploration is mandatory, as well as in patients with failed endovascular treatment. During relaparotomy, T-tube drainage of the HJ is an essential component to control ongoing biliary leakage. However, even after successful endovascular treatment, additional surgery may be required to address persistent infection or anastomotic insufficiency (Figure 4). Although surgical hemostasis was achieved in only about half of our patients, survivors of the acute phase often experienced acceptable subsequent outcomes. Taken together, these findings support an interventional-first strategy whenever technically feasible, although this interpretation should be considered in light of the limited sample size.

HIAA was associated with markedly increased mortality compared with the overall pancreatic surgery cohort. Thirty- and ninety-day mortality rates were more than sevenfold higher, with deaths attributable to hemorrhagic shock or subsequent multiorgan failure. Even among patients with HJ insufficiency, mortality was numerically higher in those who developed hemorrhage, although statistical significance was not reached, likely reflecting limited sample size.

Importantly, patients in whom primary endovascular therapy successfully achieved hemostasis demonstrated lower mortality. While causal conclusions cannot be drawn, this finding suggests that early hemorrhage control may be an important determinant of survival. This observation aligns with previous reports on DPH and reinforces the critical role of prompt diagnosis and intervention [16,21,22].

This study has several limitations that should be acknowledged. First, its retrospective design inherently limits the ability to establish causal relationships and may be subject to selection and information bias. Accordingly, the findings should be interpreted as exploratory and hypothesis-generating rather than confirmatory.

Second, although the overall cohort is large, the number of HIAA events is small. This reflects the rarity of the condition but limits statistical power and the robustness of subgroup analyses. As a consequence, the observed associations should be interpreted with caution.

Third, due to the limited number of events, multivariable analysis was not feasible without a high risk of model overfitting. Therefore, we were unable to identify independent predictors of HIAA, and all reported associations are based on univariable analyses.

Finally, treatment strategies evolved over the extended study period, which may have influenced management approaches and outcomes. Nevertheless, given the rarity of HIAA, our cohort represents one of the largest single-center experiences to date and provides clinically relevant insights into an underrecognized and potentially life-threatening complication.

## 5. Conclusions

HIAA is a rare but highly lethal complication after pancreatic surgery. It appears to represent a distinct clinical pattern characterized by delayed onset, frequent sentinel bleeding, and an association with sepsis and completion pancreatectomy. Early recognition, prompt imaging, and an interventional-first treatment strategy may improve outcomes. Larger multicenter studies are needed to confirm these findings and to better define risk factors and optimal management strategies.

## Figures and Tables

**Table 1:** Distribution of underlying diagnoses and surgical procedures in patients with hepaticojejunostomy, stratified by the occurrence of hepaticojejunostomy insufficiency-associated arterial hemorrhage (HIAA). Completion pancreatectomy was overrepresented among patients with HIAA.

Underlying Disease	Without HIAA (n = 1400)	With HIAA (n = 13)	*p*-Value
Benign diseases	491 (35.1%)	8 (61.5%)	0.075
Chronic pancreatitis	335 (23.9%)	5 (38.5%)	0.324
IPMN tumors	106 (7.6%)	3 (23.0%)	0.086
Acute pancreatitis	2 (0.1%)	---	---
Ampullary adenoma	48 (3.5%)	---	---
Malignant tumors	909 (64.9%)	5 (38.5%)	0.075
Pancreatic head carcinoma	708 (50.6%)	4 (30.8%)	0.202
Ampullary carcinoma	76 (5.4%)	---	---
Cholangiocarcinoma	99 (7.0%)	1 (7.7%)	1
Duodenal carcinoma	18 (1.3%)	---	---
Others	8 (0.6%)	---	---
Surgical procedure			
Classic pancreatoduodenectomy	83 (5.9%)	1 (7.7%)	0.574
pp-pancreatoduodenectomy	630 (45%)	5 (38.5%)	0.780
Total pancreatectomy	214 (15.3%)	2 (15.4%)	1
Completion pancreatectomy	22 (1.6%)	4 (30.8%)	<0.001
Double bypass	305 (21.8%)	---	---
Hepaticojejunostomy	104 (7.4%)	---	---
Redo-pancreaticojejunostomy	42 (3%)	1 (7.7%)	0.380

**Table 2:** Comparison of major postoperative complications between patients without and with hepaticojejunostomy insufficiency-associated arterial hemorrhage (HIAA). Cholangitis, anastomotic insufficiency, and sepsis occurred significantly more frequently in patients with HIAA.

Complication	Without HIAA (n = 1400)	With HIAA (n = 13)	*p*-Value
Cholangitis	64 (4.6%)	2 (15.4%)	<0.001
Pancreaticojejunostomy insufficiency	60 (4.3%)	7 (53.8%)	<0.001
Hepaticojejunostomy insufficiency	64 (4.6%)	13 (100%)	<0.001
Upper gastrointestinal bleeding	31 (2.2%)	1 (7.7%)	0.240
Intra-abdominal postoperative hemorrhage	13 (0.9%)	---	---
Sepsis	22 (1.6%)	6 (45.2%)	<0.001
Fascial dehiscence	11 (0.8%)	1 (7.7%)	0.060

## Data Availability

The original contributions presented in this study are included in the article. Further inquiries can be directed to the corresponding author.

## Abbreviations

The following abbreviations are used in this manuscript:

DPH	Delayed post-pancreatectomy hemorrhage
HJ	Hepaticojejunostomy
HIAA	Hepaticojejunostomy Insufficiency-Associated Arterial hemorrhage
PJ	Pancreaticojejunostomy
